# Salicylic Acid, a Multifaceted Hormone, Combats Abiotic Stresses in Plants

**DOI:** 10.3390/life12060886

**Published:** 2022-06-14

**Authors:** Junli Liu, Gaoyang Qiu, Chen Liu, Hua Li, Xiaodong Chen, Qinglin Fu, Yicheng Lin, Bin Guo

**Affiliations:** Institute of Environment, Resource, Soil and Fertilizer, Zhejiang Academy of Agricultural Sciences, Hangzhou 310021, China; liujunli16@126.com (J.L.); qedison@126.com (G.Q.); liuchen@mail.zaas.ac.cn (C.L.); lisar2002@zju.edu.cn (H.L.); cxd511@126.com (X.C.); fuql161@aliyun.com (Q.F.); lyc5918@sina.com (Y.L.)

**Keywords:** abiotic stress, reactive oxygen species, salicylic acid, signalling

## Abstract

In recent decades, many new and exciting findings have paved the way to the better understanding of plant responses in various environmental changes. Some major areas are focused on role of phytohormone during abiotic stresses. Salicylic acid (SA) is one such plant hormone that has been implicated in processes not limited to plant growth, development, and responses to environmental stress. This review summarizes the various roles and functions of SA in mitigating abiotic stresses to plants, including heating, chilling, salinity, metal toxicity, drought, ultraviolet radiation, etc. Consistent with its critical roles in plant abiotic tolerance, this review identifies the gaps in the literature with regard to the complex signalling network between SA and reactive oxygen species, ABA, Ca^2+^, and nitric oxide. Furthermore, the molecular mechanisms underlying signalling networks that control development and stress responses in plants and underscore prospects for future research on SA concerning abiotic-stressed plants are also discussed.

## 1. Introduction

Salicylic acid (SA) is a phytohormone that plays multifaceted signalling roles in mediating plant growth, development, and defences to environmental stresses [1,2]. It is a simple beta-hydroxy phenolic acid that was firstly isolated from willow, and its name was derived from the Latin word “*salix*”. The amount of SA across different plant species ranges from 0.1 to 10 µg g^−1^ fresh weight, and most SA is stored in methylated and/or glucosylated forms [3]. In plants, SA can be synthesized via two distinct enzymatic pathways: the phenylalanine ammonia-lyase (PAL) and the isochorismate synthases (ICS) pathway, which both require the primary metabolite chorismate [4]. The PAL pathway mainly takes place in the cytoplasm. Firstly, the phenylalanine is converted into cinnamic acid by PAL; then, the side chain of cinnamic acid is decarboxylated to form benzoic acid; and finally, the benzoic acid undergoes 2-hydroxylation to form SA. This pathway has been confirmed by silencing PAL genes in pathogen-attacked Arabidopsis, which resulted in a 90% reduction of basal PAL activity and exhibited a 50% decrease in SA production [5]. Another pathway mainly occurs in the chloroplasts, mediated by ICS, which directly catalyses the conversion of chorismate into isochorismate. According to this pathway, SA is generated from chorismate by the synthesis of ICS1 and ICS2 [6]. Loss of ICS1 suppresses the pathogen-induced SA accumulation in SA-deficient mutants, *sid2* [7], whereas loss of both ICS genes results in further reduction in the biosynthesis of SA [6].

SA is best known as a defence hormone. The first report on SA signalling was involved in plant immunity in 1979, which described that the application of aspirin (acetyl-SA) in virus-susceptible tobacco conferred resistance against tobacco mosaic virus [8]. During the early 1990s, studies with transgenic plants revealed how SA is perceived and synthesised under pathogen attack [9]. Recently, increasing evidence has shown that SA plays an important role in mediating plant responses to various abiotic stresses, including chilling [10], drought [11], thermogenesis [12], osmotic stress [13], and metal toxicity [14]. Although SA has been confirmed as an important signalling molecule for the regulation of reactive oxygen species (ROS) production in plants [15], most of the literature so far has found that pre-treatment with an appropriate level of SA may induce an acclimation effect on all kinds of abiotic stresses [14,16,17]. Indeed, the signalling roles of SA depend on many factors, including plant species, application mode, the exogenous and endogenous levels of SA, as well as stress faced by the plants [16]. Although recent studies have unravelled some of the molecular mechanisms’ signalling networks that control plant development and stress responses, a few regarding them are still unknown. This review starts with the literature on the role of SA in the protection of plants against abiotic stresses (including heat, chilling, salinity, metal toxicity, drought, ozone, pesticide, and ultraviolet radiation), followed by a proposal of possible mechanisms and prospects for research on SA in abiotic-stressed plants.

## 2. Functions of SA in Mitigating Abiotic Stresses

### 2.1. Heat

Global warming is causing a serious threat to plant growth and food security. Heat stress disturbs plant cellular homeostasis, retards development, and causes sterility and reduced yield [18]. It has been reported that the application of exogenous SA enhances rice yield under high-temperature conditions [19], while inhibiting the synthesis of SA markedly reduced the level of thermotolerance in pea plants [20]. Furthermore, the biosynthesis of SA was increased under heat stress, as observed in many plant species, such as mustard [21], creeping bentgrass [22], grape [23], and melon [24]. These findings indicate that SA signalling is involved in heat acclimation in plants (See Table 1).

Photosystem II, which functions as an electron transport chain in chloroplasts, is one of the most thermosensitive structures in plants [25]. A study found that spraying 0.25 mM SA onto alfalfa leaves for 5 days ameliorated the heat damage to PSII and photosynthetic efficiency [12]. This may be because SA improves the antioxidant system and chlorophyll fluorescence [26], thus maintaining the thermo-stability of the electron donor and reaction centres of PSII [27]. Heat stress also disturbs osmotic potential and destroys plasma membranes, thereby leading to ion leakage in plant cells. The application of SA can enhance free proline content, which plays a key role in the osmoregulation of plant cells. This phenomenon has been widely observed in wheat [28], cucumber [26], and tomato [29,30]. Furthermore, spraying 100 mM SA on grape leaves stabilized the activity of the proton pumps in membranes, including H^+^- and Ca^2+^-ATPase, which may be another important mechanism for maintaining the integrity of the membrane under heat stress [31]. Activities of SA contribute to better regulation of stomatal aperture along with photosynthetic apparatus, such as PSII and Rubisco activity, and thus increase the capacity of photosynthesis when subjected to stressful temperature conditions [30].

Transcriptome analysis of plants has revealed SA signalling of heat-stress-responsive genes during thermotolerance, such as NPR1 (non-expresser of pathogenesis-related), HSPs (heat shock proteins), MBF1c (multiprotein bridging factor 1c), TGA, and PR-1 (pathogenesis-related protein 1) [22,32]. Exogenous application of SA induces the synthesis of heat shock proteins (HSPs), the proteins chiefly responsible for defence against heat stress, as noted in *Arabidopsis thaliana* plants [33], tomato [34,35], and rice [36]. However, a study with transgenic Arabidopsis obtained the inconsistent results that SA failed to affected the expression of Hsp [33], indicating the molecular mechanism still needs to be further investigated. Endogenous free SA stimulated the production of PIP2-phospholipase C of pea, a lipid-associated enzyme involved in intracellular signalling, in response to heat treatment. In response to heat stress, the pea plant elevated the synthesis of SA initially, which then signalled the production of PIP2-phospholipase C, a lipid-associated enzyme involved in intracellular signalling [37]. SA also increases the expression of the chitinase-1 gene in melons under heat shock [24]. Furthermore, cross–talk between SA and other plant signallings, such as H_2_S, Ca^2+^, IAA, and ABA, has also been reported [38,39,40]. For example, treatment with SA increases the activity of L-cysteine desulfhydrase, a key enzyme in H_2_S biosynthesis, indicating that H_2_S might be a downstream signalling molecule in SA-induced heat tolerance [38].

**Table 1:** The collected references of SA on heat tolerance in plants.

SA		Heat Treatment	Plant Species	Main Responses	Reference
Type of SA	SA Treatment				
	0–0.5 mM	38 °C for 72 h	Alfalfa (*Medicago sativa* L.)	I, II, III	[12]
	0–1.5 mM	T_(max)_ ≥ 35 °C for 128 d	Rice (*Oryza sativa* L.)	I, II, III	[19]
	100 µM	38 °C for 24 h	Grapevine (*Vitis vinifera* L.)	I, II	[23]
	1 mM	40 °C for 24 h	*Cucumis sativa* L.	I, II, III, V	[26]
	100 µM	43 °C for 5 h	Grape (*Vitis vinifera* L.)	I, III, VII	[27]
	0.01%	15–35 °C for 60 d	wheat (*Triticum aestivum* L.)	I, II, III	[28]
	1 mM	42 °C for 36 h	Tomato (*Solanum lycopersicum* L.)	I, II, III, V	[29]
	0.5 mM	40 °C for 6 h	Wheat (*Triticum aestivum* L.)	I, II, III, Ethylene formation	[30]
	100 µM	45 °C for 3 h or 6 h	Grape plants (*Vitis vinifera* L.)	I, Plasma membrane H^+^-ATPase, Ca^2+^-ATPase	[31]
	10 µM	40 °C for 1 h	*etr-1*, *nahG* mutants	I, II	[32]
	0–1 mM	38 °C for 16 h	*npr1* mutant, *NahG* plants, *cpr5* mutants	I, V, VII, VI, PR genes	[33]
	0–1 mM	40 °C for 30 min	*Lycopersicon esculentum L.*	I, VII, Membrane permeability	[34]
	1 mM	41 °C for 2 h	Rice (*Oryza sativa* L.)	I, VII	[36]
	100 µM	45 °C for 3 h	Pea (*Pisum sativum* L.)	I, VI, Expression of PIP2-PLC^+^	[37]
	100 µM	44 °C for 3 h	Grape plants (*Vitis vinifera* L.)	I, II, V, PM-Ca^2+^ATPase, V-Ca^2+^ ATPase	[40]
Endogenous SA		37 °C for 2 h	Pea (*Pisum sativun* L.)	I, II, VII, VI	[20]
		45 °C for 1 h	Mustard (*Sinapis alba* L.)	I, II	[21]
		50 °C for 20 s	Melon (*Cucumis melo* L.)	I, Up-chitinase1 gene	[24]
		40 °C for o–48 h	Maize (*Zea mays* L.)	I, III, VII, VI, II, III, ABA, and IAA	[39]

I, Growth; II, Antioxidant system; III, Photosynthesis; IV, SA-induced genes; V, Electrolyte leakage; VI, Endogenous free SA; VII, Heat shock proteins.

### 2.2. Chilling

Chilling injury is one of the main limitations in the growth and productivity of tropical and subtropical crops. The regulatory role of SA in defending against chilling stress has been reported in many plant species, such as maize [41], mountain rye [42], watermelon [43], beans [44], wheat [10,45], and barley [46]. Furthermore, low temperatures induced the accumulation of endogenous SA in *Arabidopsis thaliana* and wheat plants, which further confirmed the relationship between SA and cold stress responses [45,47] (See Table 2).

Low temperatures are effective for the storage of fruits and vegetables, but they may also cause chilling injury. SA as a highly efficient buffering agent against cold stress has been widely demonstrated in many fruits. For example, spraying 0.5 mM SA changed H_2_O_2_ metabolisms and increased the chilling tolerance of banana seedlings [48,49]. Similar results have been reported in cut flowers [50], bamboo shoots [51], as well as fruits in lemons [52,53], cucumbers [54], bell peppers [55], peaches [56,57], pomegranates [58,59], and plums [60].

A study also reported that SA exposure alleviated chilling injury on the seed germination of mountain rye, musk melon, and bean plants [42,44,61]. This may be because SA activates protein synthesis, such as that of 20S proteasome, and simulates the activity of enzymes, such as pentose phosphate, gluconeogenesis, and glycolysis, which together release the plant from the quiescent state [62]. Meanwhile, Zhao et al. (2021) found that treatment with 1 µM SA alleviates chilling injury in peach fruit through enhancing the total soluble sugars together with related genes expression (SPS4, NINV2, SuSy2, and SUT1) and cold-response genes expression (DREB1A and DREB2A) [63]. SA has also been shown to play a key role in growth development (including phonological aspects such as photosynthesis, respiration, and osmolytes synthesis) under cold stress. For example, SA treatment promotes the chilling tolerance of shoots in maize, rice, and cucumber, which is accompanied by an increase in the activities of glutathione reductase (GR) and guaiacol peroxidase [64]. Spraying SA maintained photosynthesis and chloroplast construction and mitigated chilling damage in grape leaves [40]. Methyl SA alleviated cold-injury-induced oxidative damage on sweet peppers by activating the alternative oxidase [65] gene expression, the key oxidase enzymes involved in electron transfer [66]. The synthesis of soluble sugars and proline might also be involved in SA signalling in *Phaselous vulgaris* [67] because these osmolytes can maintain the composition and ratio of fatty acids in membranes due to antioxidant (form of NADH or NADPH) and osmotic adjustment [68], thus stabilizing the overall structure of cells, which is a prerequisite for cold tolerance [52,69,70]. Furthermore, it was reported that exogenous SA (1mM) promoted chilling tolerance in cucumber plants by upregulating the cold signalling pathway (ICE1, CBF1, and COR47) and genes related to SA metabolism (PAL, ICS, and *SABP*2) [71].

Proteomic studies have gradually uncovered the relationship between SA and proteins expressed under low-temperature stress. Treatment with SA has been reported to reduce ice nucleation and induce anti-freezing protein, which inhibits ice crystal formation in plant cells [72]. Treatment with 0.01 mM methyl SA induced pathogenesis-related protein expression and increased the cold tolerance of tomatoes [73]. Introduction of 2 mM SA enhanced the γ-aminobutyric acid shunt pathway of anthurium-cut flowers during storage at 4 °C, providing sufficient ATP content to cope with the oxidative damage induced by the low temperature [74]. Treatment with 2 mM SA reduced chilling injury in lemons by increasing the production of HSPs [53]. Furthermore, it was reported that exogenous SA (1 mM) improved chilling tolerance in cucumber plants by upregulating the cold signalling pathway (ICE1, CBF1, and COR47) and genes related to SA metabolism (PAL, ICS, and SABP2) [71].

However, a study using an SA-deficient mutant, *NahG*, showed a higher growth rate than wild-type plants under cold stress. After being stored at a temperature of 5 °C for a 2-month-long cultivation, *NahG* plants displayed 2.7-fold larger biomass than the wild-type plant Col-0, while the free SA levels of *NahG* were only 5% of Col-0. This indicates that SA-mediated cold tolerance still needs to be studied further, in particular, regarding SA homeostasis in plants [47].

**Table 2:** The collected references of SA on chilling tolerance in plants.

SA Treatment		Chilling Treatment	Plant Species	Main Responses	Reference
Type	Treatment				
Exogenous SA	100 µM	4 °C for 7 d	Wheat (*Triticum aestivum* L.)	II, IV	[10]
	0.5 mM	2 °C for 2 d	Maize (*Zea mays* L.)	I, II, III, Ethylene	[41]
	0–100 mg kg^−1^	10 °C for 12 h–15 h; 15 °C for 12 h	Mountain rye (*secale montanum*)	I	[42]
	0–1mM	10/5 °C for 7 d	Watermelon (*Citrullus lanatus*)	I, II, III, IV, V, SA biosynthesis	[43]
	0.1 mM	15 °C for 0–30 d	Bean (*Phaseolusvulgaris* L.)	I, II, III, Phytohormone	[44]
	0.1 mM	7/5 °C for 0– 28 d	Barley (*Hordeum vulgare*)	II, Apoplastic proteins	[46]
		5 °C for 36 d	*NahG*, *npr1* mutant, *cpr1* mutant	I, II, VI	[47]
	0.5 mM	5 °C for 3 d	Banana (*Musa acuminata coll*)	II	[48]
	1 mM	5 °C for 10 d	Banana fruits (Musa AAA group.)	I, II, V	[49]
	0–4 mM	4 °C for 21 d	Anthurium andraeanum	I, II, V, VIII	[50]
	0–3 mM	1 °C for 50 d	Bamboo shoots	I, II, V, VIII	[51]
	2 mM	−0.5– 4.5 °C for 28 d	Lemon (*Citrus limon* L.)	I, II, V, VIII	[52]
	2 mM	−0.5–4.5 °C for 28 d	Lemon (*Citrus limon* L.)	II, VII, IV	[53]
	7 mM	2 °C for 50 d	Cucumber (*Cucumis sativus* L.	I, II, III, V, VI, VIII	[54]
	200 µM	4 °C for 25 d	Pepper (*C. annuum* L.)	I, II, V, Fatty acids metabolism	[55]
	0–1 mM	0 °C for 28 d	Peach (*Prunus persica* L.)	I, II, VII	[56]
	1 mM	0 °C for 35 d	Peach fruit (*Prunus persica Batsch*)	II, Polyamine contents	[57]
	0–1.0 mM	0 °C for 0–84 d	Pomegranates (*Punica granatum* L.)	I, II, Primary metabolism	[58]
	0–2.0 mM	2 °C for 90 d	Pomegranates (*Punica granatum*)	I, II, V	[59]
	0–2.5 mM	1 °C for 0–60 d	Plums (*Prunus salicina Lindl.*)	I, II, V, Ethylene	[60]
	0–0.5 mM	20 °C for 8 d	Muskmelon (*Cucumis melo* L.)	I, II	[61]
	0.5 mM	2.5 °C for 1–4 d	Maize, Cucumber, Rice	I, II, V	[64]
	0.1 mM	0 °C for 14 d	Pepper (*C. annuum* L.)	I, IV	[65]
	0–3 mM	0 °C for 2–4 d	Bean (*P. vulgaris* L.)	I, II, III, IV, Soluble sugars	[67]
	0–5 mM	4 °C for 2 d	Acha inchi (*Plukenetia volubilis*)	II, Soluble sugars	[70]
	0–1 mM	15/10, 10/5, 5/3 °C for 0–45 d	Wheat (*Triticum aestivum* L)	I, II	[72]
	0–0.5 mM	4 °C for 2 w–4 w	Tomato (*Lycopersicon esculentum* L.)	I, IV	[73]
	2 mM	4 °C for 21 d	Anthurium cut flowers	I, II, Fatty acid metabolism	[74]
Endogenous SA		4 °C for 0–21 d	Wheat (*Triticum aestivum* L.)	Stress-protective proteins, Phytohormone	[45]

I, Growth; II, Antioxidant system; III, Photosynthesis; IV, SA-induced genes; V, Electrolyte leakage; VI, Endogenous free SA; VII, Heat shock proteins; VIII, Phenolic metabolism.

### 2.3. Salinity

When grown in saline soils, plants may suffer from superabundant ion and osmotic stress, leading to ion imbalance and toxicity in plant cells [75]. It has been found that salt stress can cause a decrease in SA content in plants, such as *Iris hexagona* [76], tomato [77], and soybean [78], whereas the application of SA increased tolerance to salt toxicity in many plant species, such as pepper [13], cucumber [79], and soybean [80] (See Table 3).

SA is an important regulator of influx and efflux of Na^+^. For instance, addition of SA to soil alleviated salt toxicity in maize by decreasing Na^+^ accumulation [81]. Exogenous foliar application of 1.5 mM SA reduced osmotic stress and improved the aerial K^+^/Na^+^ ratio of saffron under saline conditions [82]. Soaking seeds of *Leymus chinensis* in SA solution lowered osmotic damage on the plasma membrane by accumulating K^+^ and Ca^2+^ [83]. SA-signalled K^+^ accumulation might be due to the activation of H^+^-ATPase in the membrane [84], which occurs via guard cells outwardly rectifying K^+^ channel (GORK), as noted in *Arabidopsis thaliana* under salt stress [85]. The increase in Ca^2+^ influx in the cytoplasm may activate the transport system of Na^+^/H^+^ in the plasma membrane, which is mediated by the salt overly sensitive (SOS) signalling pathway [86]. Furthermore, the application of SA has been shown to maintain the membrane integrity by regulating compatible metabolites such as proline and soluble sugars. Irrigation of the solution with 1 mM SA into soil increased proline content and sustained membrane integrity of pepper cells [13]. Exogenous SA increases proline, soluble carbohydrates, and proteins contents in soybean leaves, thereby adjusting the water content of cells [84]. Pre-treatment of SA might induce a pre-adaptive response through a transient increase in H_2_O_2_ level, which may act as a second messenger to “set up” the plant to defend the following salt stress that may occur. Pre-treatment with SA enhances the activities of antioxidant enzymes in plants, which in turn decreases stress-induced oxidative stress, as has been noted in *Leymus chinensis* [83] and *Iris pseudacorus* [87]. The signalling role of SA is also cross-linked with ABA, glycinebetaine, and ethylene (ET), as they are closely correlated with the synthesis of stress proteins and maintenance of leaf water potential [88,89].

However, a transgenic investigation revealed a negative result while comparing wild-type *Arabidopsis thaliana* and an SA-deficient mutant (*NahG*) under salt stress. Wild-type plants showed extensive necrosis in shoots when grown in a 100 mM NaCl medium. Under the same conditions, *NahG* plants were more tolerant and retained green leaves [90]. Subsequent research on *Arabidopsis thaliana* transgenic mutants or lines further demonstrated that the high level of SA (*snc1*, 15 fold higher than wild-type) [91] in plants increased salt-induced damage, while a low level of SA contributed to the tolerance of plants to salt toxicity [92]. It seems as though the excessive levels of SA in plants may aggravate the oxidative burden induced by salt stress, as SA is a key signalling compound during the regulation of the antioxidant system [93,94].

The molecular basis for salinity amelioration associated with SA positively regulates the antioxidant genes, especially for GST-gene family members [16]. Pre-treatment with SA reinforced the antioxidant defence systems and mitigated the negative effects of salt stress in barley *(Hordeum vulgare* L.) [95]. Exposure of SA alleviated the salinity stress in *Solanum lycopersicum* by regulating the expression of SlGSTT2, SlGSTT3, and SlGSTF4 [94]. Exogenous application of SA improved the salt tolerance in *Triticum aestivum* due to the enhancement of transcript levels and activities of ascorbate (AsA)-GSH pathway enzymes antioxidant genes, such as GPX1, GPX2, DHAR, GR, GST1, GST2, MDHAR, and GS [93].

**Table 3:** The collected references of SA on salinity tolerance in plants.

SA Treatment		Salinity	Plant Species	Main Responses	Reference
Type	Treatment				
Exogenous SA	1 mM	0–150 Mm for 10 d	*Capsicum annuum*	I, II, III, Phenolic content	[13]
	200 µM	100 mM for 4 h	Tomato (*Lycopersicon esculentum* Mill.)	I, II	[77]
	1 mM	0–10 dS m^−1^	Soybean (*Glycine max* L.)	I, II, III, VI	[80]
	0–1.0 mM	40 Mm for 56 d	Maize (*Zea mays* L.)	I, II, III, VI	[81]
	1.5 mM	0–15 dS m^−1^ for 900 d	*Saffron*	I, II, III, VI	[82]
	0–0.5 mM	2.51 g kg^−1^ for 17 d	*Leymus chinensis Trin*.Tzvel.	I, II, V, VI	[83]
	1 mM	0–100 mM	Soybean (*Glycine max* L.)	I, III, VI, H^+^-ATPase	[84]
	50 µM	100 mM for 14 d	*rbohD* mutant, *gork1-1* mutant	I, V, VI	[85]
	0.5 mM	100 mM for 15 d	Mungbean (*Vigna radiata* L.)	I, II, III, VI, Ethylene	[88]
	0.05 mM	2% for 24 h	Wheat (*Triticum aestivum* L.)	I, II, Phytohormones	[89]
	0.5 mM	250 mM for 72 h	Wheat (*Triticum aestivum* L.)	I, II, IV	[93]
	0–0.1 mM	100 mM for 7 d	*Solanum lycopersicum*	II, GST genes	[94]
Endogenous SA		0–400 mM for 0–48 h	*Iris hexagona* walter	Phytohormones	[76]
		0–140 mM for 80 d	Soybean (*Glycine max* L.)	I, III, Phytohormones	[78]
		0–100 mM for 0–7 d	Cucumber (*Cucumis sativus L.*)	I, II, VI, Phytohormones	[79]
		100 mM for 15 d	*NahG* plants	I, II, Stress-induced genes	[90]
		0–300 mM for 14 d	*nahG*, *npr1-1*, *snc1/nahG* mutants	I, II, V	[92]

I, Growth; II, Antioxidant system; III, Photosynthesis; IV, SA-induced genes; V, Electrolyte leakage; VI, Ion uptake.

### 2.4. Metal Toxicity

Metal phytotoxicity has been a major subject of current plant biology research. Heavy metals can be absorbed easily by plant roots, transported into shoots, and cause various visible toxic symptoms, such as growth retardation, leaf chlorosis, wilting, and cell death. The beneficial role of SA in defence against metal toxicity has been reported in a wide range of plant species [96,97]. For instance, application of SA improved the growth and photosynthetic abilities in Pb-stressed rice [98], Cu-stressed *Phaseolus vulgaris* [99], and Ni-stressed mustard [100]. Recently, the co-reaction of SA with other promoters has also been evaluated. For example, combination exposure of SA and plant-growth-promoting bacteria reduced the Cr-induced oxidative damage in maize [101]. SA in combination with kinetin or calcium ameliorated Ni and Pb stress in *Phaseolus vulgaris* plants [102]. The combined supplementation of melatonin and SA effectively detoxified As toxicity by modulating phytochelatins and nitrogen metabolism in pepper plants [103] (See Table 4).

Cadmium is one of the most toxic and widespread heavy metals in the world [104]. It is the typical toxic metal that can induce representative symptoms in plants, such as replacing and inactivating essential elements, destroying protein structure, and interfering with photosynthesis, respiration, and cell division [14]. A wide range of plant species have shown that SA is deeply involved in promoting Cd tolerance during processes such as plant growth, element assimilation, Cd translocation, photosynthesis, and senescence [14]. Therefore, this review on the topic of metal toxicity is focused on the interaction of SA and Cd in plants.

The phytotoxicity of cadmium (Cd) is a major subject of current plant biology research. Recent studies have shown that the synthesis of SA in plants is markedly promoted by Cd stress. For example, after 25 μM Cd treatment, the bound SA of maize was 10 times higher than that of untreated plants [105]. Similar phenomena have been observed in barley [106] and *Pisum sativum* [107]. Studies on a wide range of plant species have shown that SA is deeply involved in promoting Cd tolerance, including in plant growth, element assimilation, Cd translocation, photosynthesis, and senescence [14].

The mediation of SA in Cd tolerance has been noted in all plant developmental stages [3]. Pre-soaking seeds with SA improved the germination rate of bluegrass and wheat when subjected to Cd toxicity [108,109]. This might be because SA upregulated protein (superoxide dismutase, NAC domain-containing protein, pathogenesis-related protein) [62] synthesis and degraded the stored proteins (storage proteins 7S, albumin 2, α-cruciferin 12S seed storage protein, aminopeptidase) [110] during seed maturation. Furthermore, SA is cross-linked with the synthesis of ABA-regulated proteins, such as dehydrins, late embryogenesis abundant proteins, and HSPs, during seed germination [62]. Furthermore, exogenous exposure to SA mitigated the inhibitory effects of Cd toxicity on growth. Pre-treatment of rice roots with 10 μM SA for 24 h significantly reversed the growth-inhibitory effect of Cd stress on day 6 compared to Cd treatment alone [81]. Cd treatment reduced the dry weight of barley seedlings by approximately 35%, whereas pre-treatment with SA significantly alleviated these inhibitory effect [106]. The effect of SA on Cd phytotoxicity is dose–dependent. No or negative effects were observed in high-SA treatments in a few plants when subjected to Cd stress, such as ryegrass [111], hemp [112], and bluegrass [109]. Furthermore, equivocal conclusions have been reported in transgenic *Arabidopsis thaliana* plant sand mutants. Excessive SA in *snc1* mutants aggravated Cd-induced inhibition, whereas SA depletion in *nahG* was mitigated by Cd toxicity [113]. However, another SA-deficient phenotype, *sid2* mutants, showed accentuated symptom when subjected to Cd stress [114].

The role of SA in Cd uptake and translocation signalling remains controversial. Foliar spraying of SA significantly decreased Cd uptake in radish plants [115]. Studies of bluegrass [109] and oilseed rape [116] also reported this finding. In contrast, the barley seedlings pre-treated with SA failed to impede Cd influx into vacuoplasts and mesophyll [106]. Studies with SA-deficient mutants, such as *sid2* and *NahG*, reported that SA did not influence Cd assimilation either in shoots or roots [115,117], and the simultaneous application of SA and Cd further increased Cd assimilation in soybean [118]. Rice roots immersed in SA experienced Cd translocation from Cd-treated parts to the Cd-untreated parts, as studied using a split-root system [119], which indicated that SA might not mediate an avoidance mechanism in Cd uptake in plants. The Cd translocation in plant roots mediated by SA might be associated with cell wall modification since the expression levels of pectin methylesterase inhibitor-encoding genes in *nahG* were dramatically higher than wild-type plants [120].

As SA plays an important role in regulating the activity of H^+^-ATPase in the plasma membrane [121], it can stabilize the optimal nutritional status of plants under Cd toxicity. Many studies have reported that SA treatment maintains the balance of ion (including K, Ca, Fe, Mn, Mg, and Zn) uptake under Cd stress, as has been reported in ryegrass [111], rice [122], bluegrass [109], and oilseed rape [116]. Treatment with SA alone may inhibit K absorption in roots [123], whereas under Cd stress, SA stimulated the K, Fe, and Mg uptake of the SA-deficient mutant, *sid2* [114]. SA is also involved in S assimilation in barley and *sid2* mutants [106,114].

The beneficial role of SA was always observed in which SA treatment was performed in advance of the Cd stress. Pre-treatment of SA can “set up” the antioxidant system and then induce the resistance. For instance, pre-treatment of SA initially increased H_2_O_2_ accumulation in rice roots. Correspondingly, the level of antioxidant system, including non-protein thiols (NPT), GSH, and ascorbic acid (AsA), and the activities of antioxidant enzymes were all elevated compared with the non-SA-exposed roots under Cd stress [119,124]. Pre-treatment of SA in acid form (SA) mainly increased the activities of antioxidant enzymes, whereas the salt form (NaSA) mainly influenced the GSH-related redox of Cd-stressed maize seedlings [125]. SA enhanced the expression levels of StSABP2, StAPX, and StSOD in potato and decreased Cd-induced oxidative damage [126]. Application of SA upregulated the expression of OsPCS1 and OsHMA3 while downregulating the OsNRAMP2 gene in Cd-exposed rice seedlings [127].

It has been reported that SA regulates plant photosynthesis through RuBisCO, redox homeostasis, light acclimation, and stomatal switch [3]. SA treatment mitigated chlorophyll destruction in soybean [128] and oilseed rape [116]. In contrast, SA deficiency aggravates Cd-induced damage on chlorophyll [114]. Under Cd stress, SA exposure significantly improved the photosynthetic yield in barley [106], increased the Fv/Fm (Fv: variable fluorescence, Fm maximal fluorescence) of melon [129], and relieved energy transfer from PSII to PSI [130]. SA application also recovered carotenoid synthesis, strengthened stomatal closure, and inhibited the activities of chlorophyll-degrading enzymes [112,131,132]. However, abnormal levels of SA also have a negative effect on photosynthesis. For example, SA deficiency upregulated the photosynthetic electron transport-related genes (PETM (phosphatidylethanolamine N-methyltransferase) and PETE1 (plastocyanin 1)) under Cd stress [133]. Pre-treatment of castor bean leaves with 500 µM SA aggravated Cd injury during photosynthesis, which might have been associated with an increase in stomatal limitation [109,134].

**Table 4:** The collected references of SA on heavy meatal tolerance in plants.

SA Treatment		Heavy Meatal Treatment		Plant Species	Main Responses	Reference
Type	Treatment	Type	Treatment			
	0.1 mM	Pb	0–0.26 mM for 18 d	Rice (*Oryzu sutivu* L.)	I, II, III	[98]
	1 mM	Cu	0–0.2 mM for 10 d	Bean seedlings	I, II, III	[99]
	0.01 mM	Ni	0–150 µM for 7 d	Mustard (*Brassica juncea* L.)	I, II, III, VIII, V	[100]
	100 µM	Cr	50 mg kg^−1^ for 7 d	Maize (*Zea mays* L.)	I, II, III, IV	[101]
	0.1 mM	Ni, Pb	2.5 mM Ni, 0.5 mM Pb for 45 d	Bean (*Phaseolus vulgaris* L.)	I, II, III, VIII	[102]
	0.5 mM	As	50 µM	Pepper	I, II, III, IV, VI	[103]
	500 µM	Cd	0–2.80 mg L^−1^ for 14 d	Maize (*Zea mays* L.)	I, II, III, IV	[105]
	500 µM		2.80 mg L^−1^ for 12 d	Barley (*Hordeum vulgare*)	I, II, III, IV, V, VI, VII	[106]
	500 µM		0–112 mg kg^−1^ for 56 d	Wheat (*Triticum aestivum* L.)	I, II, III, IV	[108]
	500 µM		0–5.60 mg L^−1^ for 7 d	Kentucky bluegrass	I, II, III, IV, V	[109]
	500 µM		2.80 mg L^−1^ for 10 d	Barley (*Hordeum vulgare*)	I, II, III, IV, V, VI, VII	[111]
	500 µM		0–100 mg kg^−1^	Hemp (*Cannabis sativa* L.)	I, II, III, IV	[112]
	300 mg L^−1^		0–1.0 mM for 40 d	Radish (*Raphanus sativus*)	I, IV	[115]
	50 µM		0–300 mg kg^−1^ for 14 d	Oilseed rape (*Brassica napus*)	II, III, V, VI	[116]
	0–0.1 mM		0–6 mg kg^−1^ for 7 d	Soybean (*Glycine max* L.)	I, III, IV, V	[118]
	10 µM		5.6 mg L^−1^ for 6 d	Rice (*Oryza sativa* L.)	I, II, IV	[119]
	0.1 mM		0–1500 μM for 15 d	Rice (*Oryza sativa* L.)	I, IV, V	[122]
	10 µM		5.60 mg L^−1^ for 6 d	Rice (*Oryza sativa* L.)	I, II	[124]
	0–500 mM		5.6 mg L^−1^ for 5 d	Soybean (*Glycine max* L.)	II, III, IV, V, VI, VII	[128]
	0–200 µM		44.8 mg kg^−1^ for10 d	Melon (*Cucumis melo* L.)	I, II, III	[129]
	10 µM		150 µM	Rice (*Oryza sativa* L.)	I, II, III	[130]
	500 µM		40 mg kg^−1^ for 6 d	Soybean (*Glycine max* L.)	I, II, III	[131]
	200 µM		11.2 mg L^−1^ for 14 d	Ryegrass (*Lolium perenne* L.)	I, II, III, VI	[132]
	0–500 µM		50 µM for 12 d	Bean (*Ricinus communis* L.)	I, III, IV	[134]
Endogenous SA		Cd	0–16.8 mg L^−1^ for 7 d	*snc1*, *npr1−1*, *nahG*, *snc1/nahG* mutants	I, II, III	[113]
			0.56 mg L^−1^ for 12 d	*Sid2* mutants	I, II, III, IV, V, VI, VII	[114]
			0.5 mM	*NahG* plants	II, IV	[117]
			50 µM for 7d	*nahG*, *npr1-1*, *snc1* mutants	I, II, VII	[120]
			5.6 mg L^−1^ for 7 d	*NahG*, *snc1* mutants	I, II, III, IV, VII	[133]

I, Growth; II, Antioxidant system; III, Photosynthesis; IV, Heavy meatal uptake; V, Ion uptake; VI, Phytochelatins; VII, SA- or heavy meatal-induced genes; VIII, Electrolyte leakage.

### 2.5. Other Stresses

#### 2.5.1. Drought

During drought stress, plants have elevated SA levels, as noted in many plant species, such as barley [135], *Phillyrea angustifolia* [136], and *Salvia officinalis* [137]. The alleviation of drought injury by SA goes along with the hardening of the antioxidant system, increasing relative water and proline contents and regulating other phytohormones [1,138]. For example, pre-treatment of SA cleared the drought-induced superoxide radical with enhancement of the expression of redox regulating genes and increased proline content with its synthesis-related genes [139] (See Table 5).

SA treatments effectively ameliorated the negative effects of drought through not only improving the photosynthetic performance and membrane permeability but also enhancing the activity of antioxidant enzymes. For instance, foliar application of SA substantially decreased the ROS and MDA contents of maize under drought stress [140]. Application of SA at 100 mM enhanced antioxidant enzymatic activities together with other physio-biochemical traits, such as membrane stability, chlorophyl content, and photosynthetic rates in wheat under drought stress [141]. When sprayed with SA at 0.5 mM, wheat seedlings effectively increased the activities of antioxidant enzymes (SOD, CAT, and PPO) to alleviate the drought-stress-induced damage effects [142]. Foliar spray of SA in sweet basil significantly promoted the plant growth and relative water contents under water-deficit conditions [143]. Spraying 2 mM SA into the leaves of *Rosmarinus officinalis* L. increased the production of essential oil under the mild drought stress (60% field capacity) [144]. Treatment with SA protected tomato plants from drought stress, mainly by maintaining membrane stability and activities of carbonic anhydrase that directly affect the rate of photosynthetic CO_2_ fixation [145,146]. Pre-treatment with SA reduced damage to the cell membranes and increased ABA content in the leaves of barley and maize, suggesting that there is cross-talk between SA and ABA during drought stress [135,147].

#### 2.5.2. Ozone

Ozone is a powerful oxidising agent that reacts with lipids and proteins in plant cells and causes oxidative damage [148,149]. SA deficiency in *NahG* plants is sensitive to the ozone, whereas ozone exposure stimulates SA accumulation and promotes virus resistance in tobacco [150]. Further evidence has shown that enhanced accumulation of SA by ozone stress is through the ICS pathway [151]. SA controls ET production of *Salvia officinalis* during ozone exposure by balancing cell redox and shrinking chlorosis formation in leaves [152]. However, abnormal levels of SA cause greater ozone injury either in deficiency or superfluousness. Many deficient genotypes, such as *Cvi-0*, *NahG*, *npr1*, *eds5*, and *sid2*, are sensitive to the ozone stress [153]. Exogenous SA application decreased the stomatal conductance, chlorophyll content, and Mg assimilation of rice under ozone stress [154]. Recently, an interesting study was conducted to test whether the O_3_-induced cell death is regulated through SA, JA, or ethylene. The global and targeted analysis of transcriptional changes in single, double, and triple mutants mainly showed that the basal SA levels are essential for plants to defend against ROS-induced cell death, which is in conjunction with ethylene and JA signalling [155,156] (See Table 5).

#### 2.5.3. Pesticide

Some chemical pesticides, such as herbicide, also directly induced the oxidative damage in plants, as observed in cucumber, pistachio plants (*Pistacia vera* L.), and barley [157,158,159,160]. The injury caused by paraquat (a kind of herbicide) continuously generates superoxide in the chloroplasts of plant cells, motivates redox reaction chains, generates various forms of ROS, and leads to oxidative damage [158]. Transgenic *NahG* in rice plants causes SA deficiency, with lower glutathione (GSH) content showing great sensitivity to paraquat exposure [158]. SA significantly increases enzymatic parameters and photosynthetic pigments of *Vigna radiata* when exposed to fungicide (mancozeb), insecticide (chlorpyrifos), and herbicide (metribuzin) [161]. Pre-treatment with 1 mM SA triggers the activity and expression of pesticide detoxification enzymes (GSTs: glutathione S-transferases; a carbon-monoxide-bound enzyme, P450 (absorption band at 450 nm)) in thiram-treated leaves [162]. Treatment with 1 mM SA promotes the degradation of pesticides and blocks their accumulation in cucumber [163] (See Table 5).

#### 2.5.4. Ultraviolet Radiation

UV radiation a key environmental signal that influences plant growth and development and can reduce disease and pest incidence [164]. However, because it is beyond the capacity of sunlight utilisation in plants, excessive exposure can directly induce the ROS production, adversely affects photosynthesis, and damage cell membranes and proteins [161]. It has been shown that SA counteracted the UV-A-, UV-B-, and UV-C-induced oxidative stress on pepper through activating antioxidant enzymes such as POD, APX, CAT, and GR [165]. Furthermore, UV radiation activated SA defences and then enhanced the tomato resistance to pathogen attack in the JA-deficient genotype [166]. Similar to ozone, UV radiation induces SA accumulation in tobacco, which is accompanied by higher activity of benzoic acid 2-hydroxylase, a key enzyme in the catalysis of SA biosynthesis [150]. It has been found that exogenous SA alleviates the damaging effects of UV irradiance in many plant species such as blue grass, soybean, and maize [167,168]. The possible roles may include increase in anthocyanin and α-tocopherol content, photochemical efficiency, and activities of antioxidant enzymes (See Table 5).

**Table 5:** The collected references of SA on other stress tolerances in plants.

SA Treatment		Stress Treatment		Plant Species	Main Responses	Reference
Type	Treatment	Type	Treatment			
Exogenous SA	1–50 mg L^−1^		10 mg L^−1^ CLO, 20 mg L^−1^ DFN, 10 mg L^−1^ DFZ; 10 d	Cucumber (*Cucumis sativus* L.)	II, III	[87]
	0.5 mM	Drought	Water deficit for 0–15 d	*Brassica napus*	I, II, Phytohormone	[139]
	0.2 g kg^−1^		Water deficit	Sweet basil (*Ocimum basilicum*)	I, II, III	[143]
	0–3 mM		Water deficit for 30 d	*Rosmarinus officinalis* L.	I, Oil compounds	[144]
	0.01 mM		Water deficit for 46 d	tomato (*Lycopersicon esculentum* L.)	II, III, V	[146]
	1 mM		Water deficit for 5 d	Maize (*Zea mays* L.)	I, II, III, ABA	[147]
	100 µM	Ozone	100–150 µg L^−1^, 5 h d^−1^, 130 d	Rice (*Oryza sativa* L.)	I, II, III, VI	[156]
	0–2 mM	Pesticide	750 g kg ^−1^ Mancozeb, 2 mL L^−1^ Termite kill, 350 g L^−1^ Anchor, for 24 h	*Vigna radiata* (L.)	I, II, III	[161]
	0–1 mM		6.6 mM thiram for 1–11 d	*Solanum lycopersicum* Mill.	I, II, III, Pesticide detoxification genes	[167]
	1 mg kg^−1^		1 mg kg^−1^ THIM, 1 mg kg^−1^ HMI, 1 mg kg^−1^ CAP	Cucumber (*Cucumis sativus L.*)	VI, Pesticides metabolism	[168]
Endogenous SA		Drought	Water deficit for 100 d	*Phillyrea angustifolia* L.	II, III	[136]
			Water deficit for 27 d	*Salvia officinalis* L.	III, JA	[137]
		Ozone	0.20 µL L^−1^ for 6 h	*Nicotiana tabacum*	IV	[150]
			0.20 µL L^−1^ for 12 h	*etr1*, *ein2*, *npr1*, *eds5*, *sid2* mutants, *NahG* plants	IV	[151]
			120 µg L^−1^, 5 h d^−1^,0–36 d	*Salvia officinalis*	I, II, III, Phytohormones	[153]
			120 µM.m^−2^.s^−1^	*NahG* plants	I, II, Cell death, Phytohormones	[154]
		Ultravioletradiation	200 µM.m^−2^.s^−1^ 24 h	*Nicotiana tabacum* L.	IV	[150]

I, Growth; II, Antioxidant system; III, Photosynthesis; IV, SA-induced genes; V, Electrolyte leakage; VI, Ion uptake.

## 3. Possible Mechanisms of SA in Mitigating Abiotic Stresses

Intensive research has shown that all abiotic stressors increase the level of endogenous SA, indicating that this simple molecule is involved in stress signalling in plants [16,169,170]. The regulatory roles of SA are mediated by various physiological processes, including growth development, photosynthesis, ion assimilation, respiration, antioxidant system, and cross-talk with other hormones [3]. The first report on the SA signalling is that it affects ROS production and then provokes pathogenesis-related1 (PR1) expression under pathogenic attack [171]. This discovery sparked the further studies on the complex signalling network between SA and ROS in plants [172]. Thus, the primary mechanism of SA reviewed here is its defensive role through redox signalling.

### 3.1. Redox Signalling

**Figure 1:**
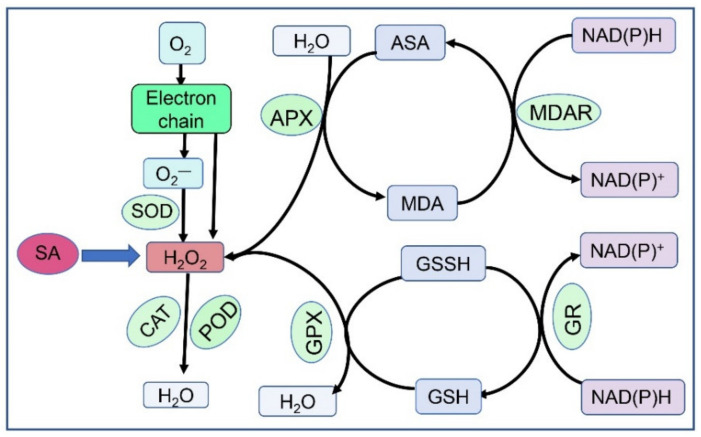
Brief pathways for reactive oxygen species scavenging in plants.

ROS are defined as the inevitable by-products of electron transfer in mitochondria, chloroplasts, and other energy-generating sites of plant cells [173]. Owing to their strong oxidisability, they can interfere with most biochemical metabolic processes, such as enzyme activity, membrane permeability, DNA stability, and protein synthesis. Under normal conditions, ROS are detoxified and maintained at equilibrium by the antioxidant defence system [174]. This system has experienced very complex evolutionary processes for 2.7 billion years. It is estimated that at least 152 genes in plants are involved in this highly dynamic and redundant network, which develops enzymatic and non-enzymatic compounds and encodes ROS-producing and -scavenging proteins [175] (See Figure 1). In many cases, the capacity to cease production of ROS is an important indicator of plant tolerance. Furthermore, low concentrations of ROS are successfully utilised by plants as a leading signalling pathway in physiological metabolic processes, such as growth development, hormone signalling, programmed cell death, cell cycle, and biotic and abiotic stress responses under normal and stress conditions [176].

Many studies have uncovered the relationship between SA and ROS in plant signal transduction. Upon pathogen attack, tobacco immediately launches the synthesis of SA, which inhibits catalase activity and leads to H_2_O_2_ burst. Then, the SA-induced H_2_O_2_ acts as a second messenger to induce defending proteins and initiate SAR [177]. Since H_2_O_2_ is an essential signal in plants against abiotic stresses, the SA-induced H_2_O_2_ may enable plants to resist subsequent abiotic stresses. This phenomenon has been especially noted in heavy metal experiments either by the modes of pre-soaking, spraying, or hydroponic exposure prior to metal stress [14]. For example, pre-treatment with SA initially induced H_2_O_2_ accumulation in rice roots, which was accompanied by an increase in the levels of antioxidant molecules and the activities of antioxidant enzymes. The strengthened antioxidant system led to a decrease in oxidative injury caused by the Cd stress [124].

During the long-term evolutionary process, plants have evolved many types of ROS-scavenging enzymes, including catalases (CATs) [175]. CATs are the most efficient H_2_O_2_-scavenging enzymes that can rapidly dismutate H_2_O_2_ into H_2_O in plant cells. Under biotic stresses, cross-talk between SA and ROS suggests that SA inhibits CAT, thus creating a self-amplifying loop of ROS production, triggering PR1 expression, and inducing SAR [171]. Detailed analyses of tobacco showed that SA acted as an electron donor for the peroxidative cycle of CAT. The self-amplifying loop of the SA-H_2_O_2_ cycle leads to the continual accumulation of SA induced by ROS in plants [15]. When SA increased to a high level (100 mM), it siphoned CAT down to the slow peroxidative reaction and then induced the damaging levels of H_2_O_2_ [9]. In mitochondria, the SA-triggered H_2_O_2_ burst occurs due to the blockage of the electron flow from substrate dehydrogenated to the ubiquinone pool [178]. Under Cd toxicity, the SA-enhanced H_2_O_2_ in rice leaves was visualised using DAB staining [179]. SA pre-treatment at an acceptable level decreased CAT activity in salt-stressed tomatoes, freezing-stressed wheat [72], and Cd-stressed rice [124]. However, this effect is not always obvious and depends on the species used, the application mode, and environmental conditions. CAT inhibition was recovered in Bermuda grass after SA exposure on day 12 under freezing stress [180]. It has also been found that SA enhances CAT activity in soybean [181] and maize [182]. Treatment with 0.5 mM SA in maize significantly inhibited the activity of the CAT1 but failed to affect CAT2 activity under chilling stress [183].

Ascorbate peroxidase (APX) is another important H_2_O_2_-scavenging enzyme that participates in the ascorbate–glutathione cycle and has a higher affinity for H_2_O_2_ than CAT. Supplementation with SA enhances the activity of APX in wheat and soybean under Cd and waterlogging stress, respectively [184]. Pre-treatment with SA did not affect APX activity of bentgrass plants after heating for the first 24 h, but after that, APX activity was maintained at a significantly higher level than that in controls [185]. It has been reported that the inhibition of APX activity is SA dependent when plants suffer a pathogen attack [186]. However, a previous study on tobacco found that SA acted on a slow-reducing substrate and did not inhibit ascorbate oxidation by APX [57].

GSH is one of the most powerful molecules in plants and plays a role in the reduction of oxidative stress and detoxification of heavy metals by chelation. Many genetic studies have reported that GSH biosynthesis is linked to endogenous SA signalling. High levels of SA were found in catalase-deficient mutants (*cat2*) together with the upregulation of GSH [187]. Compared to *cat2*, the *cat2 atrbohF* mutant with low SA content attenuated the synthesis of GSH [188]. Similar results were also found in other SA-deficient mutants, such as *sid2*, *npr1−1*, and *mpk4-1* [189,190]. Serine acetyltransferase is an important precursor enzyme that catalyses cysteine formation by GSH. A few studies with *Arabidopsis thaliana* mutants have shown that both genetics and exogenous SA increased the specific activity of SAT and GSH contents [191]. The enzymes related to GSH metabolism include glutathione synthetase (GSHS), GR [192], glutathione S-transferases (GST), and glutathione peroxidase (GPX). Endogenous SA regulates the LcGSHS transcript and leads to a higher GSH content and Cd tolerance in Cd-stressed *Arabidopsis thaliana* [193]. The synthesis of GR in *Arabidopsis thaliana* is mediated through the SA signalling pathway, and SA deficiency decreases the GR transcription of *sid2* and lowers GSH levels [194]. Photooxidative stress in the chloroplasts of *Arabidopsis thaliana* was alleviated by SA along with the depletion of GPX [195]. The expression of the promoter regions of GST genes, such as *osgstu4*, *osgstu3*, *as-1*, and *Gnt35*, was simulated by SA, suggesting that SA has class-specific functions for this enzyme [196].

Although the benefits of SA signalling have been thoroughly studied, few studies have reported ambiguous results. SA treatment mitigated Cd toxicity in barley but failed to affect the activity of antioxidant enzymes [84]. High levels of SA promote the generation of H_2_O_2_ in leaves [15]. The results of studies on SA mutants are still contradictory. High SA levels in *snc1* mutants generated a large amount of ROS, whereas SA deficiency in *NahG* lowered Cd-induced oxidative stress [91]. However, this finding is in contrast to the case of *sid2* mutants, in which Cd-induced oxidative damage was aggravated by the SA deficiency [197].

### 3.2. Cross-Talk with Other Plant Hormones

Besides of ROS, other plant hormones are involved in the SA signal transduction pathway of plants [198]. Most studies have observed a relationship between ABA and SA levels under stress. Treatment with SA induces ABA concentrations in barley and tomato [199]. Exposure of *Arabidopsis thaliana* leaves to ABA inhibits SA transduction both upstream and downstream through the SAR signalling pathway, and this suppressive effect is not related to jasmonate (JA)/ET-mediated signalling [200]. Similarly, salt stress increases the content of JA and ABA but decreases the levels of IAA, gibberellic acid (GA), and SA in *Iris hexagona* and soybean [76,78]. Insect feeding caused a strong accumulation of JA-specific mRNA transcripts, such as GmBPI1, GmKTI1, and GmAAT, but did not influence the free SA or SA-marker gene transcripts accumulation [201]. Drought stress increases the levels of SA and ABA in *Brassica napus*, and the effect on ABA is more pronounced [194]. However, the signalling role of SA might be stronger than that of ABA because the inhibition of SA biosynthesis leads to serious heating damage compared to the inhibition of ABA biosynthesis [202]. It seems that the biosynthesis of ABA is a downstream signalling event associated with SA sensing. Treatment with SA in salt-stressed tomato resulted in ABA accumulation in both root and leaf tissues together with upregulation of some ABA biosynthesis genes, such as SlZEP1, SlNCED1, SlAO1, and SlAO2 [203]. In pea plants, the activity of SA glucosyl transferase may be inhibited by ABA, thus enhancing the concentrations of free SA [204].

Calcium (Ca^2+^) is a key messenger in plants that can induce various defence responses against stress. SA-induced stomatal closure is associated with ABA signalling, and this process is mediated by Ca^2+^/Ca^2+^-dependent protein kinases (CPK) in *cpk3-2* and *cpk6-1* mutants but not in the Ca^2+^-independent protein kinase Open Stomata1 (OST1) *ost1-3* mutant [205]. It was also observed that SA triggered the Ca^2+^-sensing receptor in chloroplast thylakoid membranes of *Arabidopsis thaliana* [135]. Calmodulin, a Ca^2+^-binding messenger protein, transduces Ca^2+^ signals by binding Ca^2+^ and then modifying the target proteins. The biosynthesis of SA is regulated by calmodulin-binding-protein (CBP60g) via the activation of isochorismate synthase 1 (ICS1) [206]. Recent studies in *Arabidopsis thaliana* have shown that the SA-signalled plant immunity is associated with calmodulin-binding transcription activators (CAMTA) [207].

Similar to the function of SA, nitric oxide plays a crucial role in controlling redox homeostasis in plant responses to abiotic stresses [208]. The application of SA and SNP (NO donor) significantly improved the heat-stress tolerance of hyacinth bean and Ni tolerance of finger millet [209]. Under As toxicity, the increase in NO concentration in rice is induced by SA through the enhancement of nitrate reductase activity [210]. SA increased As tolerance in maize by activating the antioxidant defence system, but this effect was completely negated when NO synthesis was blocked [211]. Furthermore, NO may act as a downstream signalling molecule that participates in SA-signalled cell wall construction, which could impede Cd influx in Cd-stressed rice seedlings [212]. Both NO and SA are involved in the signal transduction of stomatal closure, and the increase in NO levels is dependent on SA-induced NO synthase-like enzymes [213].

### 3.3. Mitogen-Activated Protein Kinase

Mitogen-activated protein kinase (MAPK) is a type of protein kinase that is specific to the threonine and amino acids serine, which is involved in cell functions and cellular responses to a diverse array of stimuli [214]. MPK3, MPK4, and MPK6 kinases are the main mediators of plant responses to biotic and abiotic stresses. Studies on *Arabidopsis thaliana* have shown that SA is involved in transmitting MAPKs cascade signalling [215]. Compared with the wild-type, approximately 50% of the basal expression level of AtMPK3 was noted in the SA-deficient mutants with low activity of AtMPK3 [216]. A 48-kD MAPKs in tobacco was identified by SA activation since it preferentially phosphorylates myelin basic protein (MBP) [217]. Conversely, MAPK regulated the levels of SA in stressed plants [218]. It was reported that SA treatment increased the TaMAPK4 transcripts in wheat under an avirulent race of pathogen attack, whereas knockdown the TaMAPK4 gene downregulated the SA accumulation [219]. Meanwhile, StMKK1 protein negatively regulated SA-related signalling pathways in defence against pathogens in potato [220]. *Mpk4* mutant accumulated excessive levels of SA, but this was not the reason for its extreme dwarf phenotype, as knocking down the ICS1 gene (SA synthesis) did not revert mpk4-impaired growth [221]. Furthermore, the accumulation of MPK4 might also be related to SA-regulated redox homeostasis, but this mechanism is still unknown and further study [222].

## 4. Conclusions and Prospects

**Figure 2:**
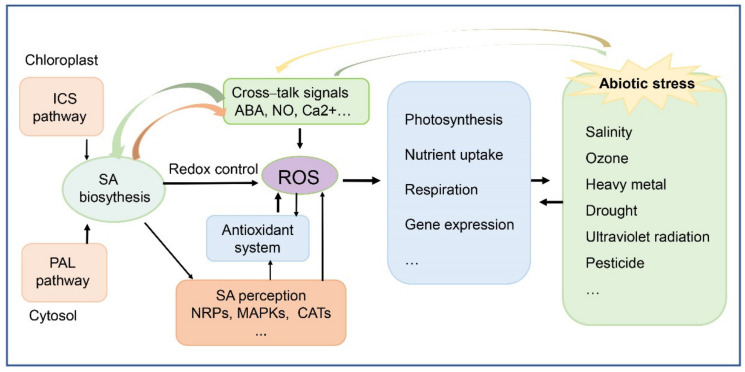
Modulation of SA signalling in plant tolerance under abiotic stress.

Increasing evidence has shown that SA acts as the primary signalling hormone in the defence against various abiotic stresses in plants. Different application modes of SA, including soaking seeds, hydroponic cultivation, and spraying on leaves, have demonstrated the protective roles. SA contributes to stress alleviation through the regulation of photosynthesis and physiological processes during growth development, and the possible mechanisms might be as follows: (1) SA indirectly regulates the antioxidant system by generating H_2_O_2_, which acts as the second messenger to induce various responses to face the abiotic stresses; (2) SA directly protects the cytomembrane and maintains the integrity of organelles by scavenging the free radicals; (3) SA takes part in the complex signal transduction network in coordination with other phytohormones, such as ABA, Ca^2+^, MAPK, and NO; and (4) under conditions of metal toxicity, SA might strengthen the cell wall and decrease the influx of heavy metals into the aerial parts (Figure 2).

Considering the complex defending roles and integration of the signalling web, dissecting the genetic network of SA in plants is a major challenge for the future studies. The transcriptome analyses coupled with metabolomic and proteomic analyses of SA in plants are essential for future studies. A few gaps exist in the signalling roles of SA under abiotic stress and understanding them requires more detailed works. For instance, how is exogenous SA absorbed by plants, and how does it affect the level of endogenous SA? How does the partial exposure of SA “set up” the whole defence system of plants? Besides SABP and NPRs, are there any other hormone receptors or gene expressions involved in the network of SA signalling pathways? How do plants accurately control the balance between endogenous levels of SA and ROS? Integration of research on SA with the development of bioinformatics tools will provide a system-level understanding of the defence roles of SA in plants under abiotic stress.

## Abbreviations

AOX	Alternative oxidase
AsA	Acetylsalicylic acid
APX	Ascorbate peroxidase
ABA	Abscisic acid
BA2H	Benzoic acid-2-hydroxylase
CATs	Catalases
Cd	Cadmium
DHAR	Dehydroascorbate reductase
DREB	Dehydration-responsive element-binding protein
ET	Ethylene
GA	Gibberellic acid
GPX	Glutathione peroxidase
GR	Glutathione reductase
GS	Glutathione synthetase
GSH	Glutathione
GORK	Guard cell outward rectifying K^+^ channel
GSHS	Glutathione synthetase
GSSH	Glutathione persulfide
GST	Glutathione S-transferases
HSPs	Heat shock proteins
ICS	Isochorismate synthases
CPK	Ca^2+^/Ca^2+^-dependent protein kinases
JA	Jasmonate
MDA	Malondialdehyde
MDHAR	Monodehydroascorbate reductase
MAPK	Mitogen-activated protein kinase
MBP	Myelin basic protein
NADPH	Nicotinamide adenine dinucleotide phosphate
NPR	Nonexpresser of pathogenesis-related
NINV2	Neutral invertase 2
POD	Peroxidase
PAL	Phenylalanine ammonia-lyase
PR1	Pathogenesis-related protein 1
PETM	Phosphatidylethanolamine N-methyltransferase
PETE1	Plastocyanin 1
SOD	Superoxide dismutase
SOS	Salt overly sensitive
ROS	Reactive oxygen species
SAR	Systemic acquired resistance
SuSy	Sucrose synthase
SPS	Sucrose phosphate synthase
SUT	Sucrose transporter
SlZEP	Zeaxanthin epoxidase1
SlNCED	9-cis-epoxycarotenoid dioxygenase
SlAO	Aldehyde oxidases
SA	Salicylic acid
SABP	Salicylic acid binding protein
